# Dropped head syndrome: report of a rare complication after multilevel bilateral cervical radiofrequency neurotomy

**DOI:** 10.1097/PR9.0000000000001037

**Published:** 2022-09-14

**Authors:** Harnek S. Bajaj, Andrew W. Chapman

**Affiliations:** a Department of Physical Medicine and Rehabilitation, Pain Medicine, Virginia Commonwealth University Health System, Richmond, VA, USA; b Department of Anesthesiology, Chronic Pain Division, Virginia Commonwealth University Health System, Richmond, VA, USA

**Keywords:** Cervical, Radiofrequency neurotomy, Radiofrequency ablation, RFA, Case report, Dropped head syndrome, Complication

## Abstract

**Introduction:**

Cervical radiofrequency neurotomy is a safe and relatively low-risk procedure commonly used to treat facet joint–mediated axial neck pain. Severe complications are extremely rare and can be avoided with proper technique and appropriate imaging guidance. This article describes the development and subsequent management of a case of dropped head syndrome after cervical radiofrequency neurotomy.

**Methods:**

A 77-year-old man with cervicalgia, multilevel facet arthropathy, and a known kyphosis in the setting of cervical degenerative disk disease underwent successful conventional radiofrequency neurotomy to the bilateral C3, C4, and C5 medial branches. No immediate complications were noted.

**Results:**

Six weeks subsequent to the procedure, the patient reported difficulty keeping his head erect, and physical examination revealed weakness of the cervical paraspinal musculature, with restriction of active extension to about neutral. A diagnosis of dropped head syndrome was made. The patient was successfully managed with temporary use of soft cervical collar and physical therapy for progressive range of motion and strengthening.

**Discussion:**

Dropped head syndrome is a known, but likely underappreciated, complication of cervical radiofrequency neurotomy, with only 2 other cases reported and published in the literature to our knowledge. Mild cases may resolve with conservative management, but this is a potentially debilitating condition that we recommend should be routinely discussed during procedural consent for cervical radiofrequency neurotomy. Future studies should explore specific mitigating factors to reduce the risk of development of this possible complication.

## Abstract 2

Dropped head syndrome is a rarely discussed complication of cervical radiofrequency neurotomy. Here, we review presentation, management, and possible mitigating factors of this complication.

## 1. Introduction

Posterior neck pain is a potentially debilitating condition with an annual global incidence of 37.2% and a lifetime prevalence of 48.5%.^7^ Cervical facet (or zygapophyseal) joint arthropathy is a common cause of axial neck pain, representing 26% to 70% of chronic cases^2,5,13,14^ and 54% to 60% of cases associated with whiplash injury.^3,10^ When the cervical facet joint is suspected as a primary pain generator, diagnosis is usually confirmed by local anesthetic block of the corresponding medial branch nerves, which innervate the zygapophyseal joints. If a positive response—usually defined as at least 80% pain relief for the duration of the local anesthetic—is obtained after 2 successive medial branch blocks, medial branch radiofrequency neurotomy (ablation) can be performed to interrupt neuronal pain signaling to the facet joints, and this procedure usually provides long-term pain relief.^11,12,15^

Radiofrequency neurotomy is a safe and relatively low-risk procedure. Transient postprocedural pain or dysesthesia is the most commonly reported complication and is usually self-limited.^9,11,15^ Although catastrophic complications such as injury to spinal nerves, ventral rami, and other unintended neuronal structures can theoretically occur, these are extremely rare if practitioners use appropriate fluoroscopic imaging guidance and avoid heavy intravenous sedation during procedures.^4,9,17^ Here, we report a case of dropped head syndrome after radiofrequency neurotomy of the bilateral C3, C4, and C5 medial branches. The patient provided HIPAA-compliant permission for the inclusion of their clinical information in this report.

## 2. Methods

**Figure 1.**
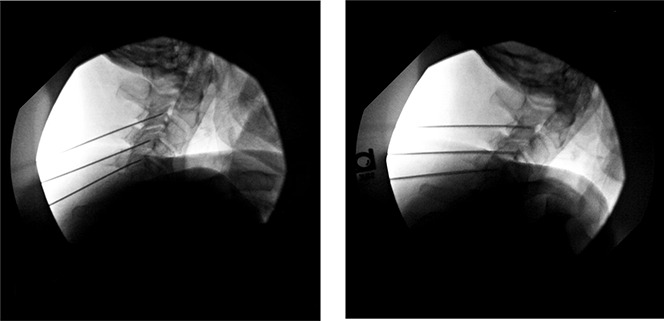
Intraoperative lateral fluoroscopic images depicting appropriate cannula placement at the right and left C3, C4, and C5 medial branches for radiofrequency neurotomy.

A 77-year-old man with a history of chronic low back pain presented with long-standing cervicalgia refractory to physical therapy and over-the-counter analgesics. Cervical spine radiographs revealed multilevel degenerative disk disease with severe disk space narrowing at C4-C5, C5-C6, and C6-C7 and facet arthropathy. The patient had a preexisting loss of vertebral height at C5 with a focal kyphosis at the C4-C5 level. Physical examination was remarkable for tenderness to palpation over cervical facets and pain with facet loading. The patient underwent 2 consecutive diagnostic medial branch blocks, 1 week apart, which resulted in temporary reduction of pain symptoms by approximately 80%. The patient then underwent conventional radiofrequency neurotomy of the corresponding medial branches of the dorsal rami at C3, C4, and C5 under fluoroscopic guidance using 16-gauge (G) cannula on the left side and 18-G cannula on the right side. Lesioning was performed at 80°C for 3 minutes (Fig. 1). The procedure was first performed on the left side, followed by the right side about one week later. Motor testing was done before thermal lesioning, and 2 lesions were created at each level. The patient reported no complications immediately after the procedures.

## 3. Results

About 6 weeks after the procedure, the patient called the clinic and reported difficulty keeping his head erect. He denied having new radicular pain symptoms or weakness in his shoulders or arms. At an office visit, the patient was noted to be holding his head in flexion. Tenderness to palpation was noted over the cervical paraspinal and upper trapezius muscles. The patient had weakness of the cervical paraspinal musculature, with restriction of active extension to about neutral, but otherwise had full strength and sensation in all myotomes and dermatomes of the upper extremities.

The patient was referred to physical therapy to work on progressive range of motion and strengthening of the cervical paraspinal muscles and was fitted with a supportive soft cervical collar for as needed use. The presumptive diagnosis was explained in detail to the patient and his spouse. A cervical MRI was ordered and showed negative results for new cord or nerve root injuries and did not definitively show abnormal signal intensity of the cervical muscles. Approximately 10 weeks later, the patient showed significant objective improvement, with the ability to actively extend the neck to 10° beyond neutral, along with improved resting position of the cervical spine. At this point, the patient discontinued the use of the cervical collar but had some residual paraspinal muscle fatigue, requiring him to ambulate with caution, using visual feedback cues for position sense. The patient has continued to work in physical therapy to improve function, muscle strength, and endurance.

## 4. Discussion

Dropped head syndrome is an exceedingly rare, and usually transient, condition resulting in a chin on chest deformity due to flexion of the head in the setting of weakness of the cervical paraspinal muscles. It is a known complication of cervical radiofrequency neurotomy, although it may not routinely be discussed during procedural consent. Patients often experience muscular neck pain and may have difficulty with eating and horizontal gaze.^6^ Dropped head syndrome is associated with several central and peripheral neuromuscular diseases and inflammatory myopathies, including Parkinson disease, myasthenia gravis, systemic sclerosis, and polymyositis.^6^ Generally, treatment consists of bracing and neck muscle strengthening exercises.^8^ Cervical fusion is reserved for refractory cases.^18^ In this case, dropped head syndrome developed in our patient after successful completion of radiofrequency neurotomy of the bilateral C3, C4, and C5 medial branch nerves of the dorsal ramus. To our knowledge, there are only 2 other cases of dropped head syndrome secondary to cervical medial branch radiofrequency neurotomy reported and published in the literature.^1,16^ In the first case, severe progressive cervical kyphosis along with the loss of all active extension of the head was noted in a patient who had radiofrequency neurotomy performed to the dorsal rami of the medial branches innervating the C2-C3, C3-C4, C4-C5, and C5-C6 levels bilaterally. After failing conservative therapy, the patient underwent C3-6 fusion with improvement in symptoms.^1^ In the second case, dropped head syndrome developed and progressed 3 months after the patient underwent left-sided radiofrequency neurotomy to the third occipital nerve and medial branch dorsal rami innervating the C2-C4 facet joints. Eventually, the patient underwent C2-T2 posterior instrumented fusion.^16^ Anecdotally, other practitioners have reported cases of dropped head syndrome that resolved with conservative management, but published literature on this condition and its management is sparse.

There were some notable differences in this case compared with those already existing in the literature. The patient had some preexisting kyphosis at the C4-5 level due to degenerative changes, which was noted on initial cervical radiographs. The patient also underwent motor testing, demonstrating local multifidus activation and absence of nerve root stimulation before radiofrequency lesioning at each level. The radiofrequency neurotomy procedures were carried out one week apart because theoretically, bilateral lesioning on the same day has been associated with greater incidence of neck muscle weakness, vertigo, and dizziness. Although unspecified in the previously mentioned cases, during our procedure, larger-gauge cannula was used (16 G, 18 G) to create a larger thermal lesion and extend therapeutic benefit. However, care was taken to position the cannula appropriately, and motor testing was performed at each level, as previously described. Fortunately, our patient retained full passive neck range of motion and the ability to achieve active neck extension to neutral post procedure. At the time of writing, about 10 weeks after the initial presentation, the patient's symptoms have continued to improve with conservative therapy, and he can actively extend his neck to 10° beyond neutral. While the precise etiology of dropped head syndrome is not completely understood, it may be related to denervation of supporting paraspinal musculature, creating a relative imbalance in the neck flexors and extensors. This would explain why the condition is often self-limited and improves with physical therapy and neck muscle strengthening. Finally, regeneration of medial branch dorsal rami is a well-known phenomenon, which usually necessitates repeat radiofrequency lesioning to treat recurrent cervicalgia. Regrowth of medial branch nerves innervating paraspinal muscles may also explain why symptom resolution can occur with time.

In conclusion, dropped head syndrome is a rare but potentially debilitating complication that may be underappreciated by spine practitioners performing cervical radiofrequency neurotomy. We recommend that this complication be routinely discussed during procedural consent for cervical radiofrequency neurotomy. One limitation of this article is we did not order electromyography. This electrodiagnostic test may prove useful in supporting a diagnosis of dropped head syndrome by detecting cervical paraspinal muscle denervation, which may not always be revealed on early cervical MRI studies. In the case of our patient, diagnosis of dropped head syndrome was self-evident based on weakness of the paraspinal musculature of relatively acute onset, his postprocedure physical examination presentation, and the absence of other comorbid neuromuscular disorders that could present with such symptoms. Future studies should explore specific mitigating factors to reduce the risk of development of this possible complication.

## Disclosures

The authors have no conflict of interest to declare.
